# Gonad Quality of Banana Shrimp Male Broodstock *Penaeus merguiensis (*De Man, 1888) Fed Different Natural Diets

**DOI:** 10.21315/tlsr2022.33.2.2

**Published:** 2022-07-15

**Authors:** Nurul Amirah Matmor, Hidayah Manan, Nor Azman Kasan, Mohamad Jalilah, Adnan Amin-Safwan, Mhd Ikhwanuddin

**Affiliations:** 1 Faculty of Maritime Studies and Marine Science, Universiti Malaysia Terengganu, 21030 Kuala Nerus, Terengganu, Malaysia; 2 Higher Institution Centre of Excellence (HICoE), Institute of Tropical Aquaculture and Fisheries, Universiti Malaysia Terengganu, 21030 Kuala Nerus, Terengganu, Malaysia; 3 STU-UMT Joint Shellfish Research Laboratory, Shantou University, Shantou, China; 4 Department of Applied Sciences and Agriculture, Tunku Abdul Rahman University College, Johor Branch Campus, Jalan Segamat/ Labis, 85000 Segamat, Johor, Malaysia

**Keywords:** Natural Diets, Sperms Quality, Broodstock, Banana Shrimp, *Penaeus merguiensis*, Diet Semulajadi, Kualiti Sperma, Induk, Udang Pisang, *Penaeus merguiensis*

## Abstract

A study was carried out to determine the maturation period, quality and quantity of sperms production in Banana shrimp male broodstock, *Penaeus merguiensis* fed different natural diets. The three different natural diets namely; squid, fish and shrimp flesh used in this study were obtained from known sources and fed to the tested shrimp in triplicate groups. Based on the results obtained, squid seem to be the most effective natural diet as it enhances the sperms maturation within 20 days. Feeding fish and shrimp flesh as diets for the Banana shrimp broodstock resulted in the observation of maturation in 22 and 24 days, respectively. Similarly, squid diet also recorded the highest (*p* = 0.002; *p* < 0.05) sperms count (58.6 to 74.5) as compared to fish diet (44.0 to 61.3) or shrimp diet (28.0 to 42.8). Also, feeding squid diet resulted in a higher percentage of live sperms with ranged between 97.75% to 98.80%. On the other hand, broodstocks fed fish and squid flesh was observed with ranges of between 96% to 97.86% and 92.54% to 96.06%, respectively. It was therefore concluded that squid diet was most effective to improve sperm quality, quantity and maturation period in male broodstock of *P. merguiensis*. Further study should be carried on the reproductive performance of broodstock fed with squid as diets and it effect on the performances of post larvae obtained.

## Abstract (ms)

Satu kajian telah dijalankan untuk menentukan tempoh matang, kualiti dan kuantiti pengeluaran sperma dalam induk jantan udang pisang, *Penaeus merguiensis* yang diberi diet semula jadi yang berbeza. Tiga diet semula jadi yang digunakan dalam kajian ini ialah sotong, ikan dan isi udang, yang diperolehi daripada sumber yang diketahui dan diberi makan kepada udang yang diuji dalam tiga salinan kumpulan. Berdasarkan keputusan yang diperolehi, sotong dilihat menjadi diet semulajadi yang paling berkesan kerana ia meningkatkan kematangan sperma dalam masa 20 hari. Induk udang pisang yang diberikan isi ikan dan udang masing-masing dilihat matang dalam masa 22 dan 24 hari. Diet sotong juga mencatatkan jumlah sperma tertinggi (*p* = 0.002; *p* < 0.05) (58.6 hingga 74.5) berbanding diet ikan (44.0 hingga 61.3) atau diet udang (28.0 hingga 42.8). Induk udang yang diberi makan sotong dilihat menghasilkan peratusan sperma hidup yang lebih tinggi di antara 97.75% hingga 98.80%. manakala, induk yang diberi makan isi ikan dan sotong masing-masing menghasilkan peratusan di antara 96% hingga 97.86% dan 92.54% hingga 96.06%. Oleh itu, disimpulkan bahawa diet sotong adalah yang paling berkesan untuk meningkatkan kualiti sperma, kuantiti dan tempoh matang dalam induk jantan *P. merguiensis*. Kajian lanjut perlu dijalankan ke atas prestasi pembiakan induk yang diberi makan sotong sebagai diet dan ia juga memberi kesan kepada prestasi larva selepas diperolehi.

**Highlights**

- Squid identified most effective natural diet to enhance the sperm maturation.
- Squid as diet also results in higher percentage of live sperms ranged between 97.75% to 98.80%.
- Squid as diet identified most effective to improve sperm quality, quantity and maturation period of male broodstock of *P. merguiensis*.

## INTRODUCTION

Shrimp, crab and lobster are three groups of crustaceans which have commercial importance in world’s fisheries industries (Pruthi 1999). Apart of that, there are more than 80 different types of shrimp species caught around the world, which are used for human consumption (Granata *et al*. 2012). According to Halver and Hardy (2002), there are evidence to support the fact that shrimp production is close to the maximum sustainable yield. Shrimp that are caught for production are unpredictable due to the uncontrollable natural phenomena on the sea. The pollution and other destructive human activities have disrupted the ecology of the shrimp nursery in the ocean. To augment the increasing market demand for this important commercial product, the aquaculture production of different species of shrimp has been intensified in the last few decades. *Commercial shrimp* culture was developed in the late of 1970s and has become a prominent component of the aquaculture industry ever since (Lim & David 1995). The world demand for shrimp is heavily inched on an increase in aquaculture production sector (Halver & Hardy 2002). Halver and Hardy (2002) reported that in 1986, shrimp cultured in ponds contributed for an estimated of 6% to 8% for the world’s total aquaculture shrimp supply. From FAO data in 1996, shrimp farming industry constituted around 43% of the estimated world shrimp consumption (Lim 1998). Nowadays, many species of marine shrimps are commercially sold and have become important aquaculture species in the industry; this includes the Banana shrimp, *Penaeus merguiensis*. According to Silvestre and Pauly (1997), a total number of 32 species of marine Penaeid shrimps have been recorded, and four of these species has become a major species of commercial importance. They are Indian prawn (*Penaeus indicus*), Tiger prawn (*P. monodon*), Green tiger prawn (*P. semisulcatus*) and Banana shrimp (*P. merguiensis*).

Marine shrimp, *P. merguiensis* had been introduced as a commercial shrimp species in Asia for a while now and can be found in Indo-West Pacific region beginning from the Persian Gulf to Thailand, Hong Kong, Philippines, Indonesia, New Guinea, New Caledonia all the way to Australia (Holthuis 1980). *P. merguiensis* shrimp is also one of the most culture species in Malaysia due to its export value (Hidayah *et al*. 2013). To compensate the high market demand, the commercial production of the *P. merguiensis* has been intensified lately. Particularly, in the aquaculture base production, great effort has been made through the development of suitable and useful techniques for culture in order to increase total harvested products on a sustainable level (Franco *et al*. 2006; Ikhwanuddin *et al*. 2019). However, production of this species is still largely affected by the low quantity and quality of sperm produced. This consequently results in the production of low numbers of live sperm, thus affecting the quality and number of offspring produced. Therefore, to increase the quantity and quality of sperm been produced, the broodstock must be fed appropriate diet with good dietary components such as squid and fish. Diet is an important factor that influences the sperm quality, quantity and maturation in shrimps. Hence, different type of natural diets with different nutrient composition may significantly affect the sperm production of the shrimp broodstock in terms of quality and quantity.

There are different natural diets that can be used for feeding the *P. merguiensis*. The widely used feed includes squid, fish, molluscs, polychaete and other decapods. Perez-Velazquez *et al*. (2002) identified that maturation diets for shrimp broodstock typically include fresh food organisms such as squid (*Loligo* sp.), bloodworms, clams and fish (*Decapterus* sp.). Jiang *et al*. (2008) also reported that the productions of sperm are dependent on culture condition and nutrition. In this study, we tested the hypothesis that the dietary component of shrimp can help stimulate the maturation, quantity and quality of sperm produced from shrimp as the objective of this study.

## MATERIALS AND METHODS

### Study Location

This study was conducted at the hatchery and laboratories of the Institute of Tropical Aquaculture and Fisheries (AKUATROP), Universiti Malaysia Terengganu (UMT), Terengganu, Malaysia. The matured Banana shrimp, *P. merguiensis* male broodstocks weighed 20 gm and above and were caught from the coastal waters of Pulau Sayak, Kedah, Malaysia (5°40’34.92” N 100°24’04.08” E) and transported to AKUATROP hatchery for further assessment.

### Experimental Design

The total of 30 mature males of *P. merguiensis* shrimps were used with 10 males for each natural diet’s treatments separated into three different tanks. The different natural diets are namely squid (*Loligo* sp.), fish (*Decapterus* sp.) and shrimp flesh (*Penaeus vannamei*) and were obtained from known sources. The feeding rate of the shrimp was at 10% of the body mass and the shrimps were fed twice a day (0800 hrs and 1700 hrs). The water used was taken from UMT coastal water, with salinity range of between 27 ppt–30 ppt. Water heater was setup to avoid fluctuation on the water temperature in the shrimp culture tank. The water in the tanks was periodically exchanged every two or three days. The tanks were provided with adequate water aeration and PVC pipe or brick stone were also included as shelter for the shrimps. A weekly sampling was conducted to identify the matured shrimp were and sample their sperm for qualitative and quantitative determination. Maturation stage and viability test examinations were done weekly to observe the quality and quantity of shrimp’s sperm.

### Viability Test

The spermatophores ejection process must be handled properly. The spermatophore was ejected carefully from the shrimp to ensure no injury to the shrimp. Improper ejection process can lead to critical injury to the shrimp, thus stressing it. The spermatophores that had been taken out from the shrimp were quickly prepared to be examined and avoid exposure to the surrounding for too long that might affect the spermatophores freshness. The spermatophores were then immediately immersed in the Calcium-free saline water (CF saline) while getting ready for the examination process. CF saline was used for maintaining the moisture of the sperm cell and preventing dryness. The percentage of live sperm is influenced by the freshness of the cell after the spermatophores is ejected from the shrimp. Feeding will not affect the percentage of the live sperm.

During the viability test, staining process is needed to ensure the visibility of the sperm cells during examination process. To observe the sperm cell of *P. merguiensis*, nigrosin and eosin staining method were applied as the stain ingredients of eosin given pink colour to the dead sperm cell instead of unstained for the live sperms.

The viability test was conducted weekly (Week 1 until Week 4) to observe the quality and quantity of sperm of the *P. merguiensis* that had been fed with different natural diets. All the tubes were filled with 100 μL of Calcium-Free (CF) saline. The spermatophores of two matured male *P. merguiensis* from each tank were taken out using forceps and transferred to the tube contains the 100 μL of CF saline. The CF saline was used to keep the moisture of the sperm, hence preventing it from dryness.

The CF saline and the spermatophores in the tubes were transferred into a petri dish. By using a dissecting microscope, the outer membrane of the spermatophores was cut off and the spermatophores was taken out. The spermatophores were transferred into a tissue homogeniser. The total of 100 μL CF saline was added into the tissue homogeniser. The spermatophores were homogenised and then 50 μL of the spermatophores that has been homogenised was taken to be inserted into a tube. The spermatophores in the tubes were stained using 25 μL nigrosin and 25 μL eosin. The stained sperm was taken out from the tube using a pipette and was placed onto a hemacytometer. The hemacytometer was observed under Advance microscope Nikon 80i (1000x). The number of live, dead and total number of spermatophores in cell/mL were then recorded.

### Sperm Maturation Determination

The shrimps from each treatment were sampled weekly to observe the maturation stage of the shrimp. The maturation stage of the shrimps was determined by observing the presence of whitish round colour of spermatophores between the fifth walking leg and the first swimming leg. The data on maturation stages observed were recorded.

### Data Analysis

Statistical analysis was conducted to analyse the collected data. The total sperm count, total number of sperm, and percentage of live sperm counted were calculated and analysed. The data analyses were done using one-way ANOVA with the statistical package SPSS 20.0. This was meant to identify if there is a significance difference between the different natural diets fed to the shrimp as it relates to the sperm maturation period, sperm quality and quantity of the *P. merguiensis*.

## RESULTS

### Sperm Maturation Period

**Figure 1:**
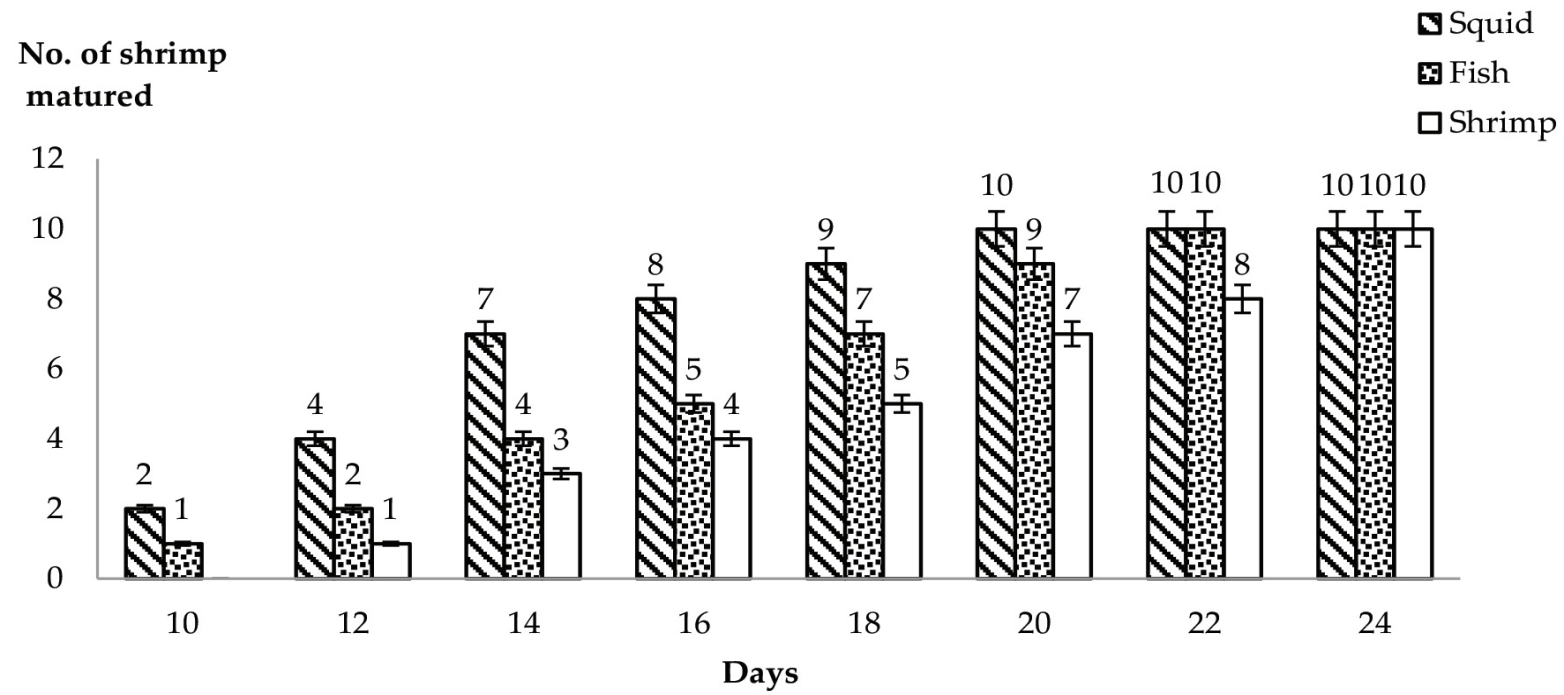
Numbers of *P. merguiensis* matured within 4 weeks study period under different natural diets condition of squid, fish and shrimp flesh. There was no significant different between diets and no. of matured shrimp where (*p* = 0.746, *p* > 0.05, *F* = 0.641) and significantly different between the days and number of matured shrimp where (*p* = 0.000, *p* < 0.05, *F* = 10.323).

The effects of different natural diets on sperm quality, quantity and maturation period of *P. merguiensis* were examined in this present study. This study revealed that *P. merguiensis* take about four weeks to completely mature. Fig. 1 showed the number of shrimps matured within the study period. Shrimp fed with squid started to become mature in Day 10. Two of the shrimps fed with squid had become matured notable with the presence of small whitish spermatophores in the bottom of their body. Only one shrimp consuming fish become mature on Day 10 and none fed with shrimp meat was observed to be mature on this day. The number of mature shrimps fed with squid increased from two on Day 10 to four (Day 12), seven (Day 14), eight (Day 16), nine (Day 18) and lastly all of 10 shrimps become mature on the 20th day of sampling. The number of mature shrimps fed with fish also increased from one (Day 10), to two (Day 12), four (Day 14), five (Day16), seven (Day 18), nine (Day 20) and lastly all 10 shrimps got matured on the 22nd day of sampling. Meanwhile, none of the shrimps fed with shrimp meat become mature in first Day 10 of the study. Only one shrimp was observed to mature on the 12th day, followed with three (Day 14), four (Day 16), five (Day 18), seven (Day 20), eight (Day 22) and lastly all 10 shrimp were matured on the 24th day. There was no significant different between the diets and number of shrimps matured where; (*p* = 0.746, *p* > 0.05, *F* = 0.6410). However, there was significant different between the days and the number of matured shrimps where (*p* = 0.000, *p* < 0.05, *F* = 10.323).

### Sperm Quantity from Total Sperm Count

**Figure 2:**
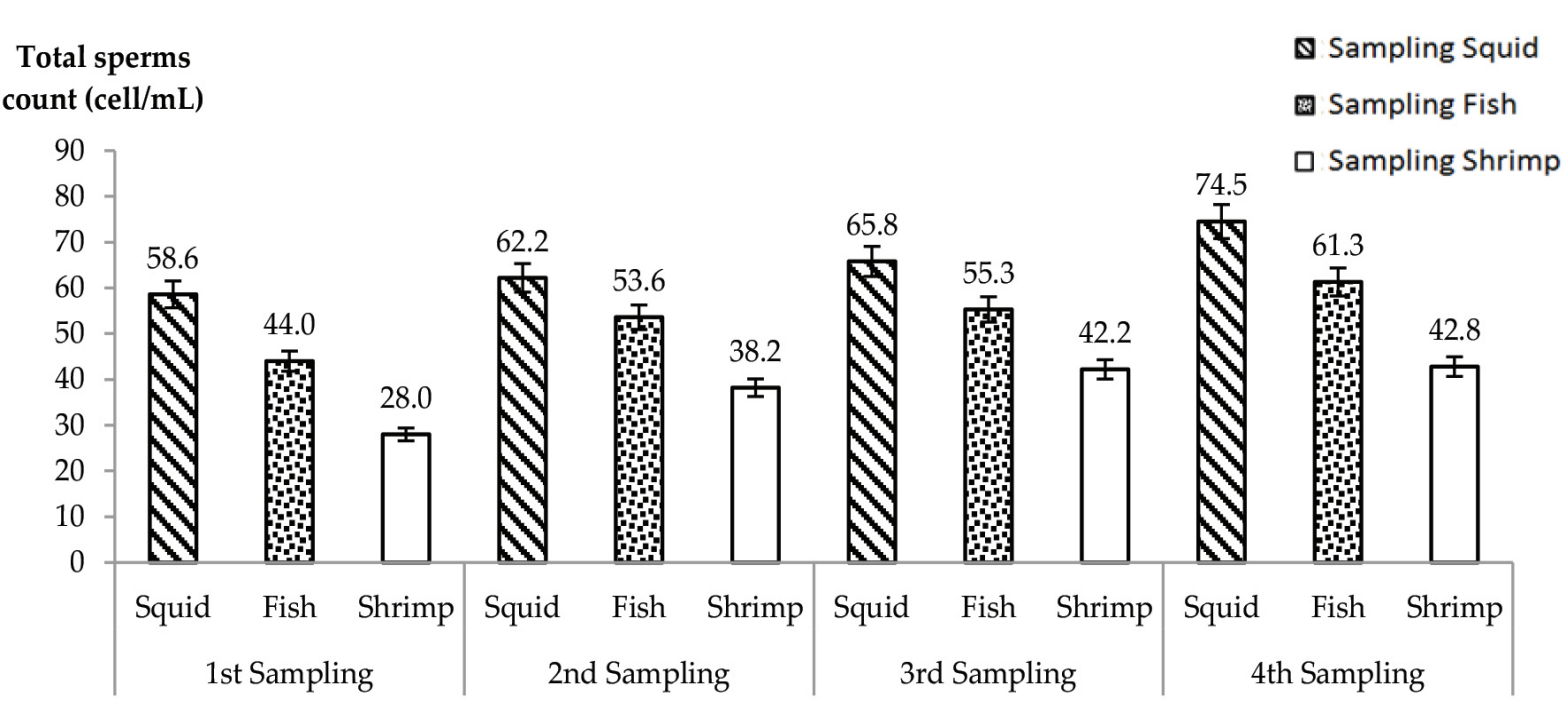
Total sperms count (cell/mL) of *P. merguiensis* for different natural diets from weekly samplings of 4 weeks study period. There was significant difference between treatments where (*p* = 0.002; *p* < 0.05). Value was presented in mean ± SD.

Fig. 2 shows the mean total sperm of *P. merguiensis* shrimp counted (cell/ mL) for each treatment as observed in the weekly sampling done. The graph shows the increases of total sperm count in *P. merguiensis* from 1st week sampling until 4th week sampling for each treatment; this means the sperm cell had increased for every week. The mean of total sperm counts in the shrimps fed with squid had increased from 58.6 ± 4.33 (1st week sampling) to 62.2 ± 3.39 (2nd week sampling) to 65.8 ± 4.02 (3rd week sampling) and lastly to 74.5 ± 3.21 (4th week sampling). For the shrimps fed with fish, the mean of total sperms count was increased from 44.0 ± 5.62 (1st week sampling) to 53.6 ± 2.95 (2nd week sampling) to 55.3 ± 3.06 (3rd week sampling) and to 61.3 ± 2.41 (4th week sampling). Meanwhile, the mean of total sperms counts in the shrimps fed with shrimp meat increased from 28.0 ± 4.24 (1st week sampling) to 38.2 ± 3.43 (2nd week sampling) to 42.2 ± 2.97 (3rd week sampling) and lastly to 42.8 ± 2.74 (4th week sampling). There was significant difference between shrimps fed with squid, fish and shrimp meat treatments (*p* = 0.002; *p* < 0.05). The result for squid treatment shows the highest mean of total sperm count, followed by fish treatment and the least was observed with shrimp flesh treatment. Based on the result, it can be seen that squid was a best diet option for the feeding of male *P. merguiensis* because it helped in stimulating the production of sperm compared to the other treatments.

### Quality of Live Sperm

**Figure 3:**
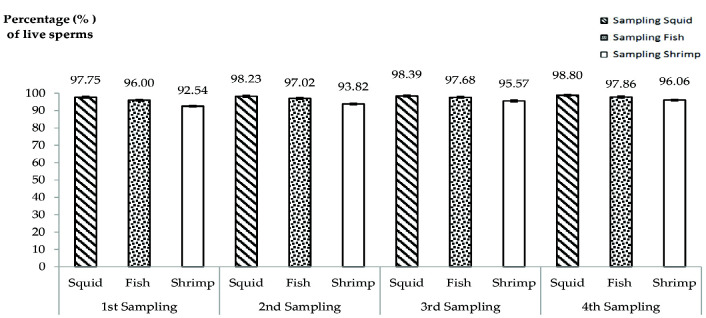
Percentage of *P. merguiensis* live sperms from different natural diet treatments from weekly sampling. No significant different between the percentage of live sperms with the first until forth samplings where (*p* = 0.669, *p* > 0.05, *F* = 0.538) and no significant different between diets with first sampling until fourth sampling where (*p* = 1.000, *p* > 0.05, *F* = 0.000). Value was presented in mean ± SD.

**Figure 4:**
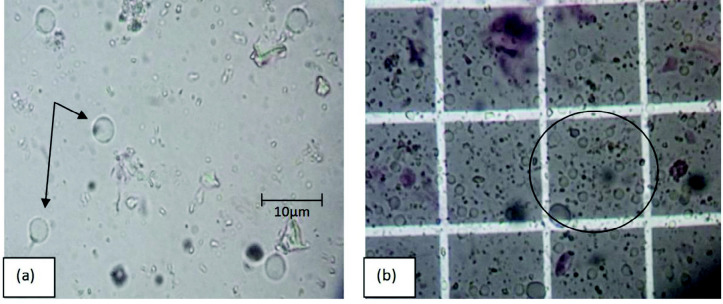
(a) The *P. merguiensis* sperms live cell view under microscope and (b) sperms cells counted by using hemacytometer, observed under 1000x magnification.

Fig. 3 shows the percentage of live sperm count for each treatment diets in every weekly sampling. In the graph, the mean of percentage of live sperm counts for shrimps fed with squid treatment shows the highest means which were 97.75% ± 2.59 (1st week sampling), 98.23% ± 2.08 (2nd week sampling), 98.39% ± 2.17 (3rd week sampling) and 98.80 % ± 1.60 (4th week sampling). The mean of percentage of live sperm count for shrimps fed with fish treatment were 96.00% ± 2.51 (1st week sampling), 97.02% ± 2.39 (2nd week sampling), 97.68% ± 1.49 (3rd week sampling) and 97.86% ± 1.40 (4th week sampling). The mean values of percentage of live sperm count for shrimps fed with shrimp treatment were 92.54% ± 3.85 (1st week sampling), 93.82% ± 3.09 (2nd week sampling), 95.57% ± 2.26 (3rd week sampling) and 96.06% ± 1.88 (4th week sampling). From analyses conducted, there was no significant different between the percentage of live sperms with the first until forth samplings (*p* = 0.669, *p* > 0.05, *F* = 0.538). There were also no significant different between diets in first sampling until forth sampling (*p* = 1.000, *p* > 0.05, *F* = 0.000). Fig. 4a showed the live sperms viewed under microscope and Fig. 4b showed the sperms cells counted using hemacytometer. All the mean percentages of live sperm count for each treatment in the graph shows a significant increment from week to week. All the mean percentages shown are slightly same. There were more than 90% and mostly near to 100% observed to be live. From the result, it shows that the percentage of live sperm is not being affected by different dietary treatments in the *P. merguiensis*. However, it is influenced by the freshness of the sperm cell, which is affected by the surrounding condition after the spermatophores were ejected from the shrimps.

## DISCUSSION

In this present study, three different types of natural diet namely squid, fish and shrimp flesh were used to stimulate the gonad production and maturation of Banana shrimp, *P. merguiensis* broodstock. All diets were proven to stimulate the shrimp’s maturation within 2 to 3 weeks. This might be due to the high level of protein in the diet that helps stimulate the shrimps to become mature. According to Braga *et al*. (2010) fish and squid have the highest level of protein, followed by crab and commercial diet. Feeding rate and feeding frequency also can influence the maturation process; 10% of feeding rate per body mass and feeding frequency of twice per day can helps stimulate the shrimp to mature. If feeding rate is lower than 10% body mass, it wills slower the maturation process. But, if the feeding is more than 10% of body mass, it will be wasteful as it results to excess feeding, which cause the food to become rotten, hence affecting the water quality. Poor water quality will give stress to the shrimp and cause mortality. Poor water quality also can affect the reproductive quality as hypothesised in many previous studies (Braga *et al*. 2010; Cavalli *et al*. 1998; Pascual *et al*. 1998, Perez-Velazquez *et al*. 2001).

All of prepared diets used to feed the shrimps were kept frozen. This is to ensure the food given remain fresh to avoid feeding rotten feeds. After every feeding, the uneaten feed inside the water must be cleaned and taken out from the tank. Water quality should be maintained to prevent the released of ammonia that can be toxic to the shrimp. The water quality must be monitored frequently. The water parameter was therefore checked regularly in this study to maintain the water quality in good condition. The water in the tanks also was exchanged regularly. The salinity of the water was maintained between 27 ppt–30 ppt. The water can be exchange for every two to three days depend of the water quality level. Poor water quality will cause stress to the shrimp and can prevent the shrimp from maturing. Jaganmohan and Kumari (2018) found out that adequate water quality plays an important role in preventing stress of the shrimp, hence, preventing their susceptibility to various diseases. In the same vein, associated water quality problems can also cause delayed maturation period and thus affect the production of sperm in the shrimp. This assumption is supported by an earlier study reported by Callan and Laidley (2010) where they observed that water chemistry is an important factor that can affect the health, reproduction performance and egg quality of *C. loricula* broodstock. It can also lead to the shrimp mortality.

From the study, squid treatment showed the best result among the three treatments, followed by fish and shrimp flesh. The shrimp fed with squid had better sperms production and faster maturation period compared to the other natural diets. According to Pascual *et al*. (1998), the spermatophores renewal completes every two or three weeks. In this present study, the spermatophores renewal in all treatment takes about four weeks to be completed, hence, becoming completely matured. This is in line with a previous study reported by Memon *et al*. (2012). It may be decreased by in proper captivity conditions such as nutritional factors and undesirable physiochemical parameters (Memon *et al*. 2012; Leung-Trujillo & Lawrence 1987; Ceballos-Vázquez *et al*. 2004). According to Samuel *et al*. (1999), nutritional factors may be the reason for the incapability of males kept under captive conditions. Thus, nutritional factors might have a considerable role in spermatophores production and maturation.

The shrimp were fed with squid; fish and shrimp flesh as diets. According to Chong and Sasekumar (1981), preference for animal food could be related to the animal’s high protein requirement. The diet used to feed the shrimp in this present study is known to have high level of protein that can meet the shrimp protein requirement. Protein is needed by shrimp for their growth (Maguire & Hume 1982) and for development of reproductive performance. The optimum protein levels required for the prawn were estimated in the range of 34%–42% for diets of energy content between 2.9 Kcal/g–4.4 Kcal/g (Chong & Sasekumar 1981; Sedgwick 1979; Slattery & Palmer 2008).

There might be a significant difference in types of food that is used for feeding. Samuel *et al*. (1999) identified that a good reproductive performance, spermatophores morphology and colour was observed in prawn fed with clam, squid and pellet food as against prawn fed with clam daily. Based on the findings of the present study, we showed that there is a significantly difference between the diets used to feed the shrimp. Shrimp fed with squid give better reproductive performance and high quantity of sperm production, followed by fish and shrimp flesh diet. Therefore, squid is suggested to be the main nutritional diet for male shrimp *P. merguiensis* broodstock.

## CONCLUSIONS

Squid as natural diets was identified as the most effective diets to improve the sperm quality and quantity also enhances the maturation period in male broodstock of *P. merguiensis*. Further study should be carried out in the future on the reproductive performance of broodstock that fed with squid as diets to assess on the effect on the performances of post larvae obtained.
